# Can buccal infiltration of articaine replace traditional inferior alveolar nerve block for the treatment of mandibular molars in pediatric patients?: A systematic review and meta-analysis

**DOI:** 10.4317/medoral.24726

**Published:** 2021-09-25

**Authors:** Jianfeng Yu, Siyan Liu, Xiao Zhang

**Affiliations:** 1 Department of Stomatology, affiliated Hospital of Shaoxing University, P.R. China; 2 Department of Implantology, Shanghai Ninth People's Hospital, College of Stomatology, Shanghai Jiao Tong University School of Medicine; National Clinical Research Center for Oral Diseases; Shanghai Key Laboratory of Stomatology and Shanghai Research Institue of Stomatology, China

## Abstract

**Background:**

It is unclear if buccal articaine infiltration can be used as an alternative to standard inferior alveolar nerve block (IANB) for treating mandibular molars in pediatric patients. Therefore, this study aimed to pool evidence to compare the efficacy of buccal infiltration of articaine vs IANB with lignocaine for pediatric dental procedures.

**Material and Methods:**

We searched the PubMed, Embase, ScienceDirect, CENTRAL, and Google Scholar databases for randomized controlled trials (RCTs) comparing the two techniques in pediatric patients and reporting the success of anesthesia and/or pain during treatment. PRISMA guidelines were followed.

**Results:**

Seven RCTs were included. Pooled analysis of five studies indicated no statistically significant difference in the success rates of the two anesthetic techniques (OR: 1.02; 95% CI: 0.13, 7.96; I2=69%, *p*=0.98). Meta-analysis of data from the four studies demonstrated no statistically significant difference in pain during the procedure with buccal infiltration of articaine or IANB with lignocaine (SMD: 0.62; 95% CI: -1.37, 0.12; I2=88%, *p*=0.10).

**Conclusions:**

Evidence suggests that buccal infiltration of articaine is a viable alternative to IANB with lignocaine in pediatric patients for treating mandibular molars. Based on the confidence intervals, there may be a tendency of higher success rates with buccal infiltration of articaine.

** Key words:**Lignocaine, articaine, primary dentition, children, molars.

## Introduction

The importance of adequate pain control for pediatric dental procedures cannot be understated. Inadequate anesthesia can lead to anxiety and fear in pediatric patients which can hamper any future dental treatment (1). One of the commonly used drugs for management of pain during dental procedures is local anesthetics and of the many local anesthetics available in clinical practice, lignocaine is the gold standard drug against which other anesthetic agents are compared (2).

Articaine is an amide anesthetic with an ester group which was initially introduced in Germany in 1976. On account of the dual feature, the drug is metabolized by hydrolysis in plasma as well as by microsomal enzymes in the liver (3). The dual metabolism reduces the half-life of articaine to only 20-40 minutes with a lowered risk of systemic toxicity with higher drug doses (3,4).

Whilst the physiochemical properties of articaine are similar to other local anesthetics, the unique feature of the drug is the presence of a thiophene ring which replaces the traditional benzene ring and improves the liposolubility of the drug. This property significantly enhances the penetration of articaine into tissues (3).

Traditionally, the inferior alveolar nerve block (IANB) is used for pulp therapy or extraction procedures for mandibular molars in both adults and children. However, IANB is associated with several disadvantages especially in children, like large area anesthetized, longer duration of anesthesia, the incidence of soft tissue injury, etc. In this context, there is growing interest in the ability of articaine to anesthetize mandibular molars using infiltration anesthesia alone (5). There have been several reports in literature demonstrating the superiority of articaine vis-à-vis lignocaine for oral anesthesia. Research suggests that the higher tissue penetration property of articaine can lead to better success rates with articaine infiltration as compared to lignocaine. When used for IANB, articaine has demonstrated superior anesthetic efficiency compared to lidocaine for mandibular third molar extractions in adult patients (6). Singular buccal infiltration injections of articaine have also been successfully used for dental extractions of maxillary and mandibular teeth in adult patients (5).

The use of articaine has also received attention in pediatric dentistry, albeit in a limited manner. In a systematic review and meta-analysis of six studies, Tong *et al* (7) have suggested equal efficacy of articaine and lignocaine for pediatric dental procedures. Contrastingly, in another recent review, Taneja *et al* (8) have indicated that articaine may have better efficacy as compared to lignocaine in pediatric patients. It is important to note that these systematic reviews have pooled evidence from studies comparing infiltration with infiltration, IANB with IANB, as well as infiltration with IANB. Therefore, it is not clear if buccal infiltration of articaine can be used instead of traditional IANB with lignocaine for pediatric patients. Since there is an absence of level-1 evidence on this important question, we hereby designed and conducted the current study to compare the anesthetic success and efficacy of buccal infiltration of articaine as compared to IANB with lignocaine for the treatment of mandibular molars in children.

## Material and Methods

- Inclusion criteria

Using the PICOS (Population, Intervention, Comparison, Outcome, Study design) framework we constituted the following inclusion criteria:

Population: pediatric patients (<18years) undergoing any procedure (restorative or extraction) of mandibular molars (primary or permanent).

Intervention: Buccal infiltration anesthesia with articaine

Comparison: IANB anesthesia with lignocaine.

Outcomes: Success of anesthesia and/or pain during treatment.

Study Design: In order to provide high quality evidence, only randomized controlled trials (RCTs) were eligible to be included.

No language restriction was placed for the inclusion of trials. Studies comparing only buccal infiltration or only IANB with either drug were excluded. We also excluded studies on adults, non-RCTs, retrospective studies, single-arm studies, and studies not reporting relevant data.

- Search Strategy

The review as conducted as per the guidelines of the PRISMA statement (Preferred Reporting Items for Systematic Reviews and Meta-analyses) (9) and the Cochrane Handbook for Systematic Reviews of Intervention (10). However, the review was not registered on any online database. An electronic search was conducted by two reviewers (S.L & X.Z), independent of each other, for the following databases: PubMed, Embase, ScienceDirect, CENTRAL, and Google Scholar. The time limit was from the inception of databases to 1st September 2020. The terms used for the literature search included: “articaine”, “lignocaine”, “lidocaine”, “buccal infiltration”, "nerve block", "pediatric", "children", and "molars". Search terms were used in different combinations to find relevant articles. After the deduplication of articles, the search records were analyzed by their titles and abstracts separately by the two reviewers. Articles matching the inclusion criteria were identified and full texts of these were extracted. Individual studies were then assessed for final inclusion in the study. Any disagreements were resolved by discussion. After completion of the search and identification of included studies, the bibliography of included articles was hand searched for any other potential article.

- Data extraction and quality of included studies

The following data were extracted from the included trials by two authors independently (S.L & X.Z): names of first authors, publication year, study type and location, age of participants, type of procedure, sample size, details of study and control drugs, pain scale used, study outcomes, and study conclusions. The primary outcome of our analysis was the success of anesthesia. The secondary outcome was pain during treatment.

We used the Cochrane Collaboration risk assessment tool for assessing the quality of included RCTs (10). Two reviewers independently assessed each study (S.L & X.Z). The following seven domains were used for quality assessment: random sequence generation, allocation concealment, blinding of participants and personnel, blinding of outcome assessment, incomplete outcome data, selective reporting, and other bias. The study was judged to have a "high", "unclear", or "low" risk of bias for each domain. Any disagreements were resolved by discussion.

- Statistical analysis

“Review Manager” (RevMan, version 5.3; Nordic Cochrane Centre [Cochrane Collaboration], Copenhagen, Denmark; 2014) was used for the meta-analysis. Outcome data was fed into meta-analysis software and cross-checked for correctness. Data on the success of anesthesia was summarized using Odds ratios (OR) with 95% confidence intervals (CI). The generic inverse variance model of the meta-analysis software was used to pool the pain scores. Standardized mean difference (SMD) were calculated for pain scores as different scales were used by the included studies. We used a random-effects model to calculate the pooled effect size for all our analysis. Heterogeneity was assessed using the I2 statistic. I2 values of 25-50% represented low, values of 50-75% medium, and more than 75% represented substantial heterogeneity. As less than 10 studies were included in the meta-analysis, funnel plots were not used to assess publication bias. A sensitivity analysis was performed by excluding one study at a time in the meta-analysis software itself to assess if any trial had an undue impact on the effect size.

## Results

**Figure 1:**
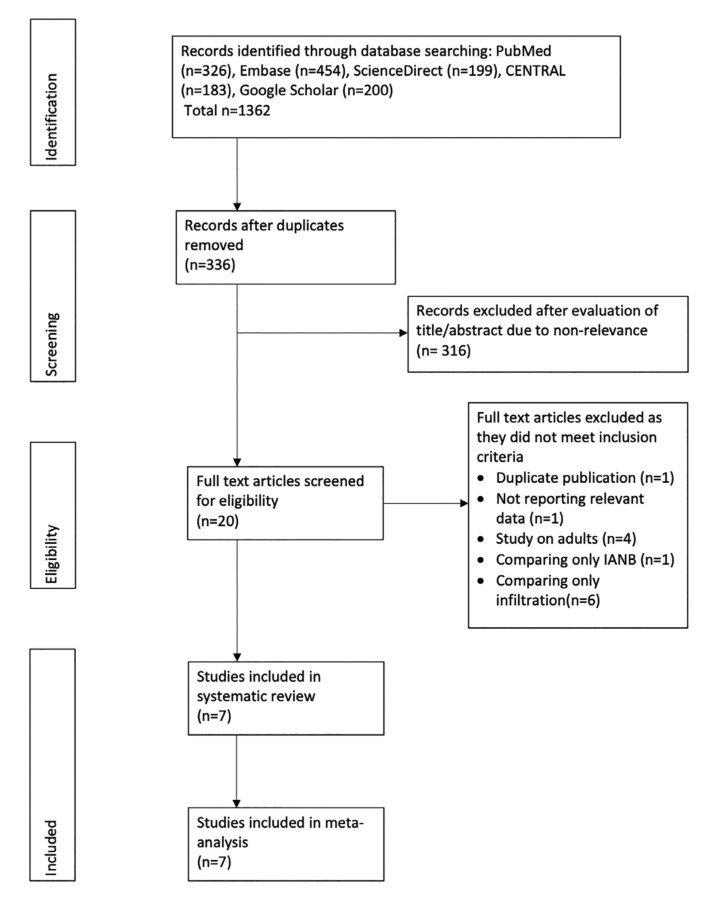
PRISMA flowchart of the systematic review.

Fig. 1 represents the PRISMA flow-chart of the study.

A total of seven RCTs fulfilled the inclusion criteria and were analyzed in our review (11-17). Thirteen studies were excluded due to the following reasons: one study each for reporting duplicate data, not reporting relevant outcome, and comparing only IANB (18). Others were conducted only on adults (19-22) or were comparing only infiltration techniques (23-28).

Details of the included studies are presented in Table 1.

**Table 1:**
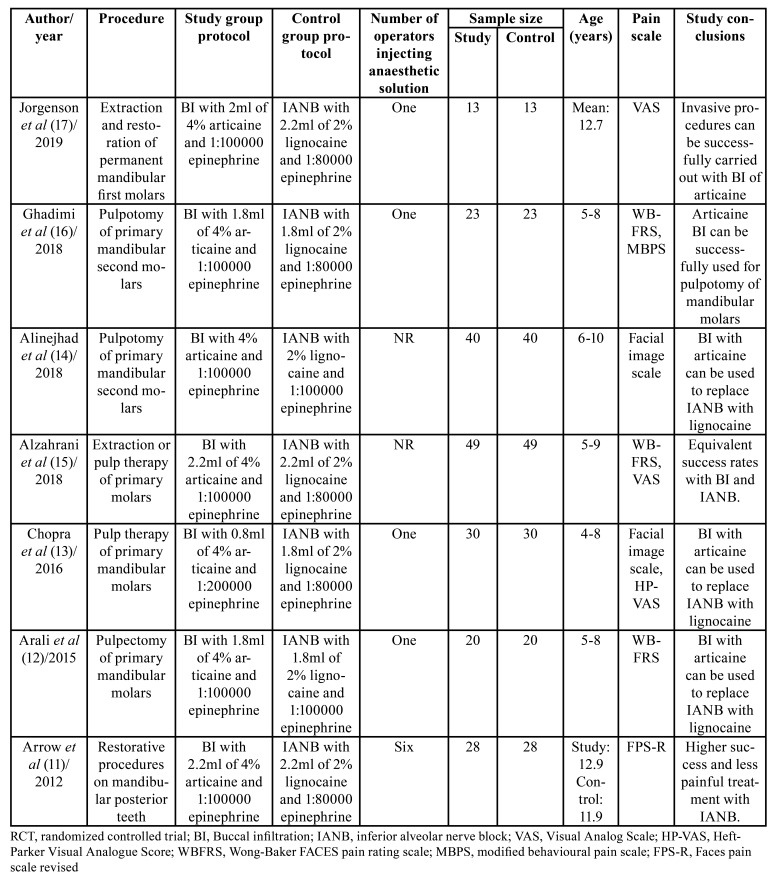
Characteristics of included studies.

Three trials were cross-over RCTs (13,14,16). Five trials were conducted on exclusively primary teeth (11-16). Studies assessed the efficacy of the anesthetic agents for both extractions and restorative procedures. Different pain scales were used across studies. Except for the study of Arrow *et al* (11), all trials reported equivalent efficacy of buccal infiltration of articaine and IANB with lignocaine for procedures on mandibular molars. None of the trials were funded by any pharmaceutical companies.

**Figure 2:**
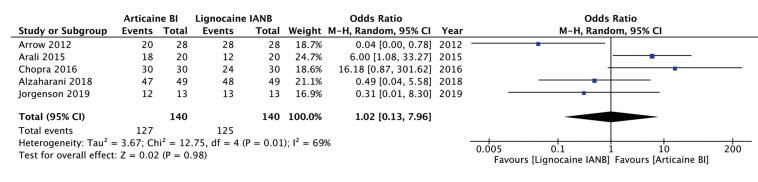
Forest plot for the success of anesthesia with buccal infiltration of articaine vs IANB with lignocaine.

**Figure 3:**
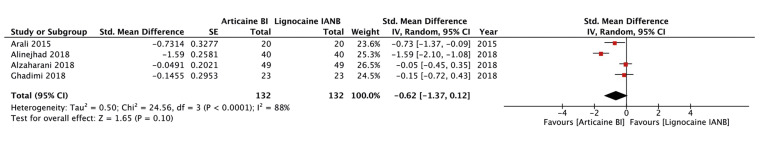
Forest plot for treatment pain scores with buccal infiltration of articaine vs IANB with lignocaine.

The success of anesthesia was reported by five of the seven included studies. Pooled analysis indicated no statistically significant difference in the success rates of the two anesthetic techniques (OR: 1.02; 95% CI: 0.13, 7.96; I2=69%, *p*=0.98) (Fig. 2). The significance of the results did not change on exclusion of any study during the sensitivity analysis. For the secondary outcome, i.e. pain during the procedure, data on a continuous scale was reported by six studies. However, two trials reported only mean pain scores without standard deviation values. Emails to corresponding authors for missing data did not elicit a response. Therefore, the results of these two studies are presented descriptively. Chopra *et al* (13) reported their data separately for males and females. The study reported significantly lower pain scores with articaine infiltration as compared to lignocaine IANB in both sub-groups (Mean scores: Males, 6.83 vs 26.5 and females, 3.28 vs 18.5; *p*<0.01 for both). On the other hand, in the second study of Jorgenson *et al* (17), the authors reported no statistically significant difference in treatment pain scores with articaine or lignocaine (14.54 vs 17.08 respectively, *p*=0.48). On pooled analysis of data from the remaining four studies demonstrated no statistically significant difference in pain during the procedure with buccal infiltration of articaine or IANB with lignocaine (SMD: 0.62; 95% CI: -1.37, 0.12; I2=88%, *p*=0.10) (Fig. 3). The significance of the results did not change on exclusion of any study during the sensitivity analysis.

**Figure 4:**
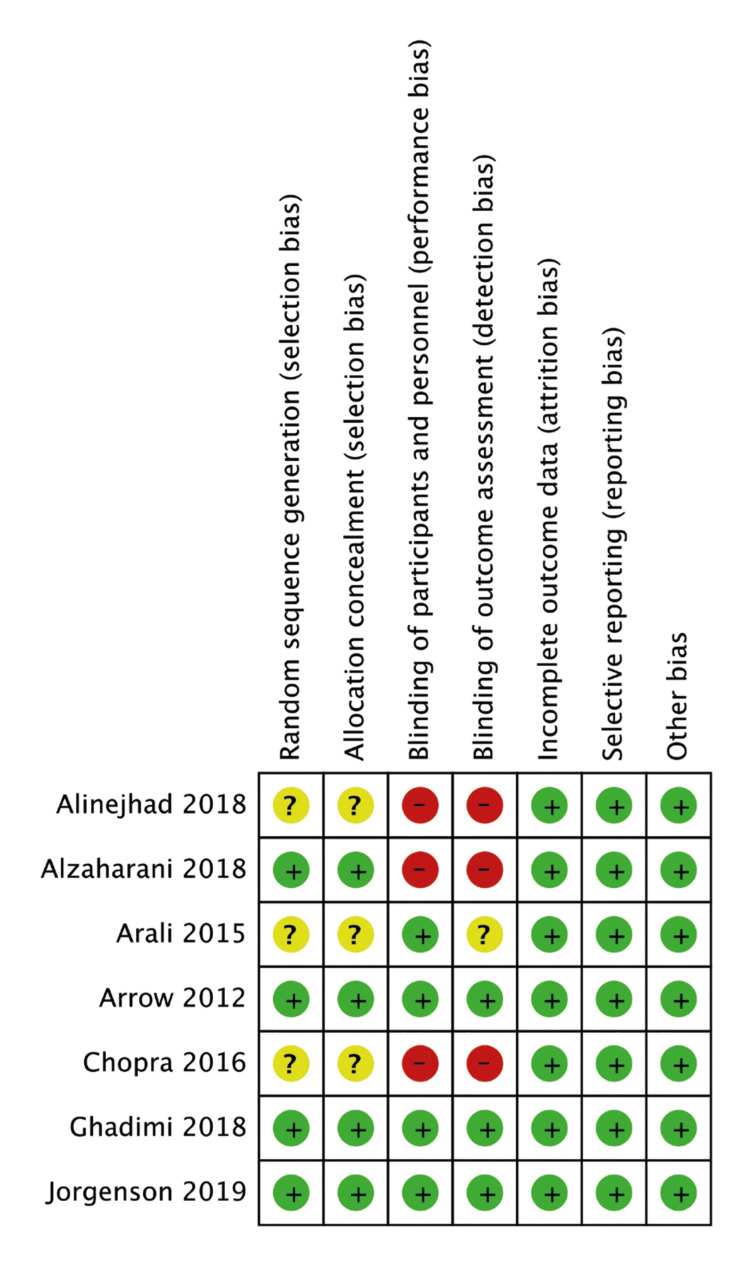
Authors' judgment of risk of bias in included studies. Green, low risk; Yellow, unclear risk; Red, high risk.

The risk of bias summary of included studies is presented in Fig. 4. According to the author's judgment, appropriate methods of randomization and allocation concealment were reported by four RCTs (11,15-17). Blinding was not carried out in three studies (13-15).

## Discussion

The results of the first systematic review and meta-analysis comparing buccal infiltration of articaine and IANB with lignocaine in pediatric patients presents two important findings: 1) There seems to be no difference between the two techniques for the success of anesthesia 2) Pain during the procedure may not be significantly different between either method.

While local anesthetics are invaluable in managing pain during pediatric dental procedures, there are instances of failure with these drugs as well. In a study involving 361 children undergoing different dental procedures, the failure rate of local anesthetics was found to be as high as 11.6% (29). Continuation of treatment with inadequate anesthesia in children can lead to uncooperative behavior and more importantly, failure of anesthesia may be misdiagnosed as uncooperativeness leading to unnecessary referrals for general anesthesia (30). Optimal pain relief in children can be achieved either with infiltration techniques or nerve blocks. Owing to the thickness of the cortical bone, infiltration with lignocaine does not provide complete anesthesia for the treatment of mandibular molars and the IANB has been the standard technique for anesthetizing patients undergoing extractions or pulpal procedures for these teeth. However, unlike infiltration anesthesia, IANB is associated with failure rates of up to 15-20% (31). This has been attributed to several factors like anatomical variations, anxiety, technical errors, etc. Secondly, the large area anesthetized with IANB is often discomforting and unnecessary given that anesthesia of only a small area is needed. Soft tissue injury due to accidental biting of the lower lip is common due to IANB especially in the pediatric cohort. Furthermore, occasional and rare complications associated with IANB like trismus, hematoma, nerve paresthesia may additionally limit the application of this technique if a more optimal anesthetic method is available (32).

The utility of buccal infiltration of articaine for anesthetizing mandibular molars has been explored owing to the high tissue penetrability of the drug. The presence of thiophene ring and intermolecular hydrogen bonds greatly enhance penetration of articaine molecule in the nerve sheath and may be responsible for better anesthetic efficacy of articaine (4). Robertson *et al* (33) in a double-blind RCT have demonstrated that 4% articaine infiltration has a significantly better success rate (75-92%) for inducing pulpal anesthesia in mandibular first molars as compared to infiltration anesthesia with lignocaine (45-67%success). Similar significant results have been noted by Nydegger *et al* (34). In this context, there may be a role of articaine infiltration for treating mandibular molars, especially in children, which may lead to the omission of IANB and its associated disadvantages.

On a systematic review of the literature, we found seven RCTs evaluating the question of interest in this review. On pooled analysis of data, there was no statistically significant difference in the success of anesthesia with either of the two techniques. The pooled OR was 1.02 but the upper end of the 95% CI was 7.96 indicating a possibility of approximately x8 times higher success rates with articaine infiltration vs lignocaine IANB. The SMD of treatment pain scores between the two methods was also insignificant indicating both techniques are capable of inducing optimal anesthesia. Even in the studies not included in the meta-analysis, pain in the articaine infiltration group was not higher as compared to the IANB group. The results of our analysis are supported by several studies in adults comparing the two techniques for treating mandibular molars. Zain *et al* (35) in an RCT on adult patients with irreversible pulpitis of mandibular molars have reported better success rates with articaine infiltration as compared to lignocaine IANB (76.9% vs 62.8%). The authors found no statistically significant difference in pain scores with the two techniques and concluded that articaine infiltration may be a viable alternative to lignocaine IANB. Bataineh *et al* (36) in a split-mouth RCT of 52 adult patients requiring permanent mandibular first molar extractions reported no difference in pain perception between infiltration anesthesia with articaine and IANB with lignocaine.

The results of our review should be interpreted with the following limitations. Firstly, there were concerns of bias due to randomization, allocation concealment and blinding in the included RCTs which may have skewed the results of the trials. Secondly, the total number of studies in the analysis was not very high. Not all of the seven studies were pooled for a meta-analysis. Thirdly, different pain scales were used by the included RCTs. This was partly offset with the use of SMD to pool data of pain scores. An important factor in assessing pain in children is that pain scores can be subjective and influenced by factors like age, gender, anxiety, presence of prior pain, and experience of dental procedures (29). The influence of these factors on our study results cannot be negated. Lastly, inter-study heterogeneity in the included studies for patients' age, treatment type, the dosage of anesthetic agents, the number of operators involved, etc. could have influenced outcomes.

To conclude, within the limitations of our review, our results indicate that articaine infiltration may be a viable alternative to IANB with lignocaine for treating mandibular molars in pediatric patients. Based on the confidence intervals, there may be a tendency of higher success rates with buccal infiltration of articaine as compared to IANB with lignocaine. The results of our study have important clinical implications as only a single and dependable buccal infiltration injection may be used for treating mandibular molars in pediatric patients thereby avoiding the need for IANB and its associated errors and complications. Future studies should compare the efficacy of the two techniques for specific treatment procedures and in different age-group to elicit more comprehensive evidence. An ideal study for the future would be a high-quality multicentric RCT with a large sample size comparing articaine infiltration with IANB separately for dental extractions and pulpal procedures in a population stratified by different age groups.
